# Correcting for the study bias associated with protein–protein interaction measurements reveals differences between protein degree distributions from different cancer types

**DOI:** 10.3389/fgene.2015.00260

**Published:** 2015-08-04

**Authors:** Martin H. Schaefer, Luis Serrano, Miguel A. Andrade-Navarro

**Affiliations:** ^1^Systems Biology Research Unit, Centre for Genomic Regulation – European Molecular Biology Laboratory, BarcelonaSpain; ^2^Universitat Pompeu Fabra, BarcelonaSpain; ^3^Institució Catalana de Recerca i Estudis Avançats, BarcelonaSpain; ^4^Faculty of Biology, Johannes Gutenberg University of MainzMainz, Germany; ^5^Institute of Molecular Biology, MainzGermany

**Keywords:** protein–protein interactions, study bias, network analysis, degree distribution, cancer genes

## Abstract

Protein–protein interaction (PPI) networks are associated with multiple types of biases partly rooted in technical limitations of the experimental techniques. Another source of bias are the different frequencies with which proteins have been studied for interaction partners. It is generally believed that proteins with a large number of interaction partners tend to be essential, evolutionarily conserved, and involved in disease. It has been repeatedly reported that proteins driving tumor formation have a higher number of PPI partners. However, it has been noticed before that the degree distribution of PPI networks is biased toward disease proteins, which tend to have been studied more often than non-disease proteins. At the same time, for many poorly characterized proteins no interactions have been reported yet. It is unclear to which extent this study bias affects the observation that cancer proteins tend to have more PPI partners. Here, we show that the degree of a protein is a function of the number of times it has been screened for interaction partners. We present a randomization-based method that controls for this bias to decide whether a group of proteins is associated with significantly more PPI partners than the proteomic background. We apply our method to cancer proteins and observe, in contrast to previous studies, no conclusive evidence for a significantly higher degree distribution associated with cancer proteins as compared to non-cancer proteins when we compare them to proteins that have been equally often studied as bait proteins. Comparing proteins from different tumor types, a more complex picture emerges in which proteins of certain cancer classes have significantly more interaction partners while others are associated with a smaller degree. For example, proteins of several hematological cancers tend to be associated with a higher number of interaction partners as expected by chance. Solid tumors, in contrast, are usually associated with a degree distribution similar to those of equally often studied random protein sets. We discuss the biological implications of these findings. Our work shows that accounting for biases in the PPI network is possible and increases the value of PPI data.

## Introduction

Protein–protein interaction (PPI) networks are important models of the functional organization of the cell. To date many small and large scale studies exist mapping PPIs in human (the integrated database HIPPIE; Schaefer et al., 2012, hosts PPIs from 34,625 different studies). However, we are still far from the complete knowledge of the human interactome (Venkatesan et al., 2009), especially when its (spatial and temporal) dynamics and context-dependence are taken into account (Ideker and Krogan, 2012; Schaefer et al., 2013). High error rates associated with the experimental methods applied to measure PPIs have been recognized as a major burden for completing this goal (Von Mering et al., 2002). However, besides experimental error, other biases pose problems on the analysis of PPI networks.

Protein–protein interaction networks are associated with two types of biases: technical biases caused by limitations inherent to the experimental techniques applied to generate the PPI networks and study biases driven by the research interests guiding the selection of bait proteins tested for interaction partners. Examples for technical biases are the tendency of tandem affinity purification followed by mass spectrometry (TAP/MS) to detect interactions between highly abundant proteins (Von Mering et al., 2002; Björklund et al., 2008; Ivanic et al., 2009) and interactions involving small proteins under 15 kDa (Gavin et al., 2002). Yeast two-hybrid (Y2H) tends to detect interactions between protein pairs located in the nucleus (Jensen and Bork, 2008).

The study bias arises due to the fact that proteins are studied an uneven amount of times: some proteins (e.g., with higher biomedical relevance) are studied more often than proteins with unknown biological function. In yeast, the more GO terms a protein is annotated to the more likely it is to be studied (Gillis and Pavlidis, 2011; Gillis et al., 2014). This type of bias is particularly strong in aggregated networks (Gillis et al., 2014) as are commonly used in network biology. Not surprisingly, highly studied proteins are associated with a higher number of known PPI partners (their degree; Hakes et al., 2008). This poses a major challenge on the analysis and interpretation of PPI networks: it might misleadingly suggest a correlation between the biological relevance of a protein and network properties as, for example, the degree of a protein. Indeed, several studies reported a higher degree for essential proteins (Coulomb et al., 2005) and for disease proteins such as cancer proteins (Wachi et al., 2005; Jonsson and Bates, 2006; Rambaldi et al., 2008). It is unclear to which extent the reported higher degree of disease proteins reflects biological properties of disease proteins in networks and how much their degree is influenced by the fact that disease proteins are studied more often than other proteins.

The observation that disease proteins have more interaction partners than non-disease proteins led to numerous computational studies using directly or indirectly the degree of a protein as a predictor for its function or disease relation (e.g., Xu and Li, 2006; Nie and Yu, 2013) that thereby might only reveal highly studied proteins that are more likely to be associated to the studied function anyway.

To avoid misleading conclusions from biased PPI networks, it was repeatedly proposed to rely on non-biased large scale screens for the analysis of network properties of distinct protein classes (Zotenko et al., 2008; Rolland et al., 2014). However, the experimental coverage of the protein set of interest is usually low when only a single or few large scale studies are considered. To our knowledge, there is only one study that addressed the bias directly with a normalization strategy for the analysis of properties of HIV targets (Dickerson et al., 2010).

Here, we first aim to quantify the impact of the study bias on the observed degree distribution in a large integrated PPI network. We then investigate if one of the most frequently made claims with respect to network properties of disease proteins, the higher degree of cancer proteins, holds when we take into account the higher number of times these proteins have been tested for PPI partners. Surprisingly, we find that a much more complex picture of the degree-disease relation emerges when correcting for the study bias, with a high heterogeneity across different cancer types.

## Materials and Methods

### Protein–Protein Interaction Data

Protein–Protein Interactions were retrieved from HIPPIE version 1.5 (Schaefer et al., 2012). HIPPIE is an integrated PPI resource aggregating all PPIs from various expert-curated databases. HIPPIE implements a confidence score, which reflects the amount and type of evidence supporting an interaction (such as the number of studies reporting an interaction). However, for the purpose of this analysis we considered all 122,755 PPIs in HIPPIE as we reasoned that filtering for experimental evidence would further increase the study bias in the resulting subnetwork. Bait usage statistics were extracted from the PPI databases Mint (Chatr-aryamontri et al., 2007), IntAct (Kerrien et al., 2007), and iRefWeb (Turner et al., 2010). We annotated the number of studies in which a protein was used as a bait.

### Statistical Analyses

Statistical hypothesis testing was performed with the *R* statistical computing environment. For estimating the significance of the Pearson correlation, the test statistic was based on Pearson’s product moment correlation coefficient. The confidence interval was based on Fisher’s *Z* transform. The randomization test was performed by replacing each cancer protein by a non-cancer protein that had been equally often tested as a bait. To obtain reasonably distinct random protein sets we included proteins with similar bait usage when there were fewer than four proteins that had been tested as a bait equally often. Therefore, we successively extended a random set with similarly often studied proteins until the size of the set exceeded four proteins. First, we included proteins tested as baits 20 times more or 20 times less often than the original protein. If there were still less than four proteins in the range we successively increased the range to 150 times tested and then to 250 times tested more or less than the original proteins.

### Cancer Data

A recent study analyzed almost 5000 different human cancer exomes and their matched normal-tissue samples to detect significantly mutated genes in a representative selection of 21 tumor types under a unified statistical framework (Lawrence et al., 2014). From this study, we extracted the enrichment of somatic point mutations for each gene and tumor type. We considered a gene a cancer gene if the enrichment *q*-value (the false discovery rate adjusted equivalent to the *p*-value) was below 0.1 for the respective tumor type. From the 21 different cancer types, we analyzed 15 that were associated with at least seven genes.

### Gene Ontology Enrichment

For the GO term enrichment analysis we used the tool ConsensusPathDB (Kamburov et al., 2011). For the analysis of highly studied proteins, only terms below a *q*-value threshold of 0.01 were considered. For the analysis of functions associated with highly connected cancer genes, we applied the same *q*-value threshold to select terms enriched among all genes of the respective cancer and additionally tested for the resulting terms if they were significantly more associated with highly connected proteins (as compared to lowly connected).

## Results

### Highly Studied Proteins have More Protein Interaction Partners

**FIGURE 1:**
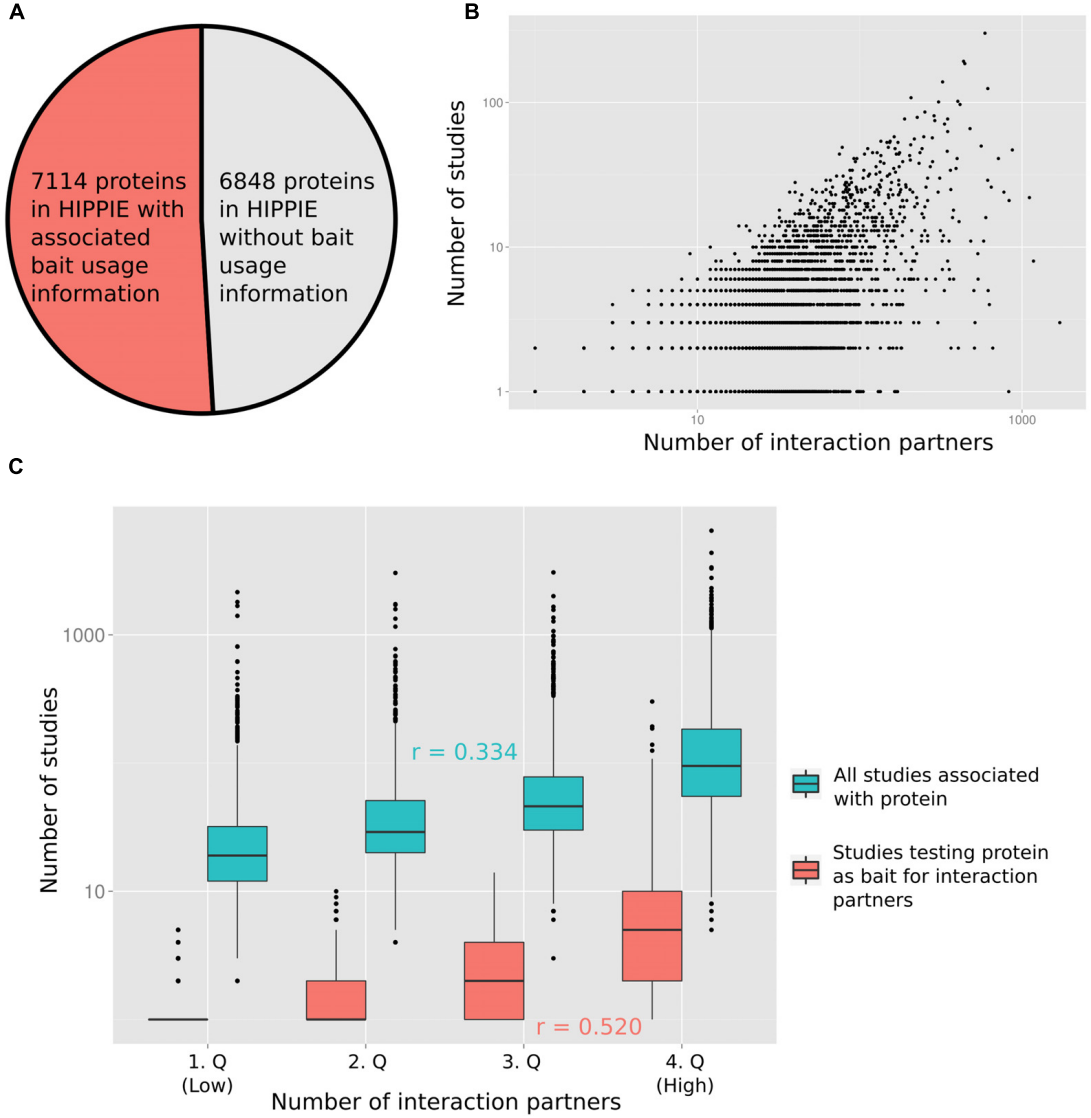
**(A)** For slightly more than half of the proteins in HIPPIE we had information of how often they had been tested as bait proteins for interaction partners. **(B)** The number of studies in which a protein has been tested as a bait for interaction partners is positively correlated with the number of reported interactions (Pearson correlation of 0.520). **(C)** The protein–protein interaction (PPI) degree distribution has been split into four equally sized quartiles (1.Q–4.Q). The distribution of numbers of all studies and only those studies testing the associated proteins as baits for interaction partners are shown for the quartiles. Pearson correlation values (*r*) are indicated.

To quantify the relation between the number of times a protein has been studied and the reported number of PPI partners, we computed the degree of each protein from the integrated PPI database HIPPIE (Schaefer et al., 2012). Next, we recorded how many times each protein has been studied as a bait in studies reporting PPIs (**Figure 1A** displays the fraction of proteins for which we had information on how often they had been tested as bait proteins). Finally, we annotated the number of PubMed abstracts linked to each protein (as a proxy for the number of studies reporting the protein; provided by the PubMed FTP server; downloaded on January 8, 2015). In **Figure 1B** the number of interaction partners of a protein is plotted against the number of studies in which the protein has been tested for interaction partners (**Figure 1B** shows the relation in log–log space, **Supplementary Figure S1** on linear scale). **Figure 1C** visualizes the relation between the number of interaction partners and both the number of all studies and of studies testing the protein as a bait for interaction partners after grouping the number of interaction partners into quartiles. As expected, the correlation between the number of times a protein has been tested for interaction partners as a bait protein and the interaction degree of a protein (Pearson correlation of 0.520) is higher than the correlation between the total number of times a protein has been studied (including studies not focused on PPIs) and the degree (Pearson correlation of 0.334). However, both variables are significantly correlated with the protein interaction degree (*p* < 10^-16^; see Materials and Methods).

### Properties of Highly Studied Proteins

Using ConsensusPathDB (Kamburov et al., 2011) we evaluated the enrichment of functions and pathways in the set of 7114 bait proteins in terms of *q*-values (see Materials and Methods for details). In accordance with a previous study that investigated functional categories enriched among entire networks (Futschik et al., 2007), we found a strong enrichment of proteins with nuclear localization, or with functions in cell cycle and metabolism (*q* < 10^-4^) among the proteins used as baits. When calculating the enrichment of functional terms and pathways among the 173 proteins most frequently used as a bait (at least 20 times) relative to that of the full bait list, most strongly enriched were “pathways related to cancer” (*q* < 10^-39^). Other strongly enriched protein classes were related to viral infection [Hepatitis B (*q* < 10^-28^), Epstein–Barr (*q* < 10^-21^), HIV (*q* < 10^-17^), and Herpes simplex (*q* < 10^-15^)] and signaling pathways [TNFalpha (*q* < 10^-28^), TGFbeta (*q* < 10^-24^), and Leptin signaling (*q* < 10^-23^)]. While the enrichment of nuclear proteins in the entire bait set might be caused by a technical detection bias of the still predominantly used Y2H assay, which requires nuclear localization of the bait and prey proteins, the strong enrichment for cancer pathways in the frequently studied bait set clearly indicates a selection bias toward proteins with high biomedical relevance.

### Correcting for the Bait Usage Bias

**FIGURE 2:**
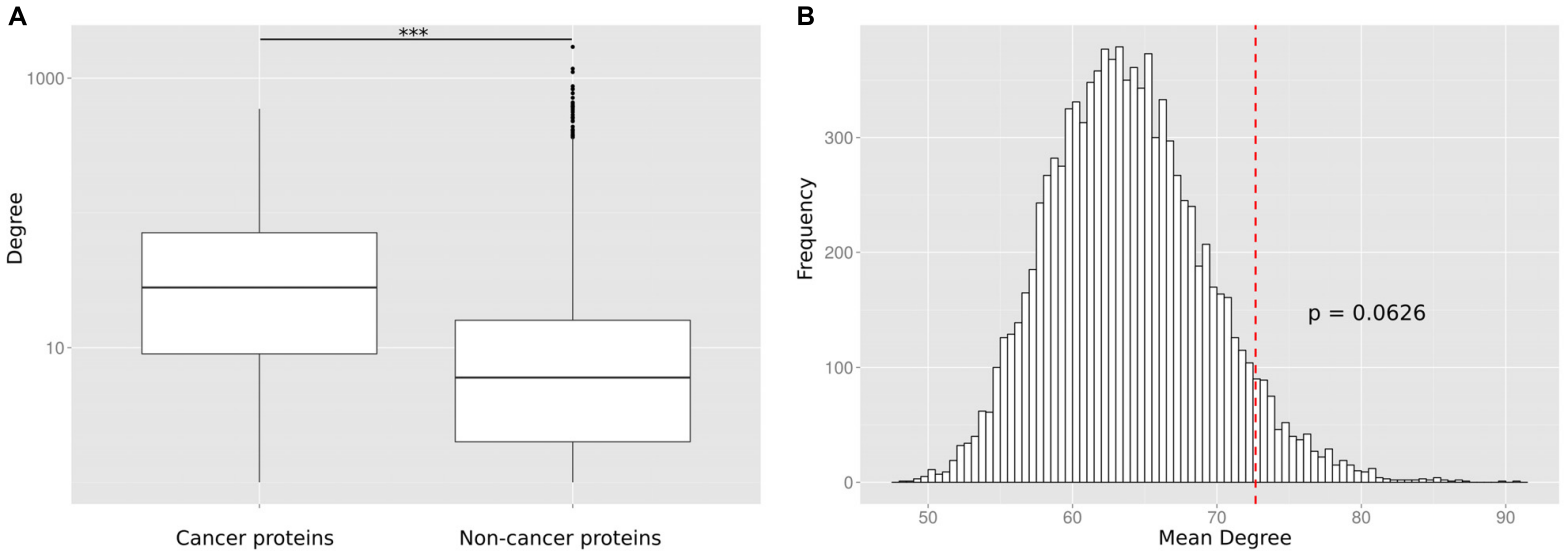
**(A)** Without correcting for the study bias the PPI degree difference between cancer proteins and non-cancer proteins is highly significant (^∗∗∗^ *p* < 10^-16^). **(B)** The number of PPIs is not significantly enriched for cancer proteins as compared to equally frequently studied random protein sets. The histogram shows the mean degree of 10,000 random gene sets with the same bait usage distribution as the cancer protein set. The position of the red dotted line indicates the mean degree of the cancer protein set. The *p*-value is computed as the fraction of times the mean degree of the randomized set was larger than the observed mean degree and is not significant (*p* = 0.0626).

To reconfirm the previously reported (Wachi et al., 2005; Jonsson and Bates, 2006; Rambaldi et al., 2008) difference in the degree distribution between cancer and non-cancer proteins, we retrieved and pooled somatically mutated cancer genes from 21 different tumor types (Lawrence et al., 2014). We compared the number of PPIs of cancer proteins to the number of PPIs of non-cancer proteins. We observed that the cancer proteins have a significantly higher number of PPI partners (*p* < 10^-16^; Wilcoxon Mann–Whitney test; **Figure 2A**) but we suspected that this difference could be an artifact caused by the largely different number of times the two protein classes have been studied for interaction partners.

To investigate this artifact, we randomly generated sets of non-cancer proteins equivalent (in terms of having been studied as baits) to the set of cancer proteins. This was done by replacing each cancer protein by a randomly selected protein used the same number of times as a bait protein than the cancer protein (or similar number of times if no protein existed that was tested the exact same number of times). For each of the 10,000 generated random sets, we calculated the mean number of interaction partners (**Figure 2B**). We found that cancer proteins tend to be involved in more PPIs than non-cancer proteins used as baits as often as cancer proteins. However, we did not observe a significant difference (a *p*-value computed as the fraction of times the mean degree of the randomized set was larger than the observed mean degree for cancer proteins; *p* = 0.0626). The lack of a significant difference between cancer proteins and equally often studied random protein sets (as compared to the highly significant difference between cancer proteins and all non-cancer proteins) suggests that previous observations on particular network characteristics of cancer proteins are biased by the differential research interest in disease versus non-disease proteins.

### Studying the Degree Distributions of Different Cancers

**FIGURE 3:**
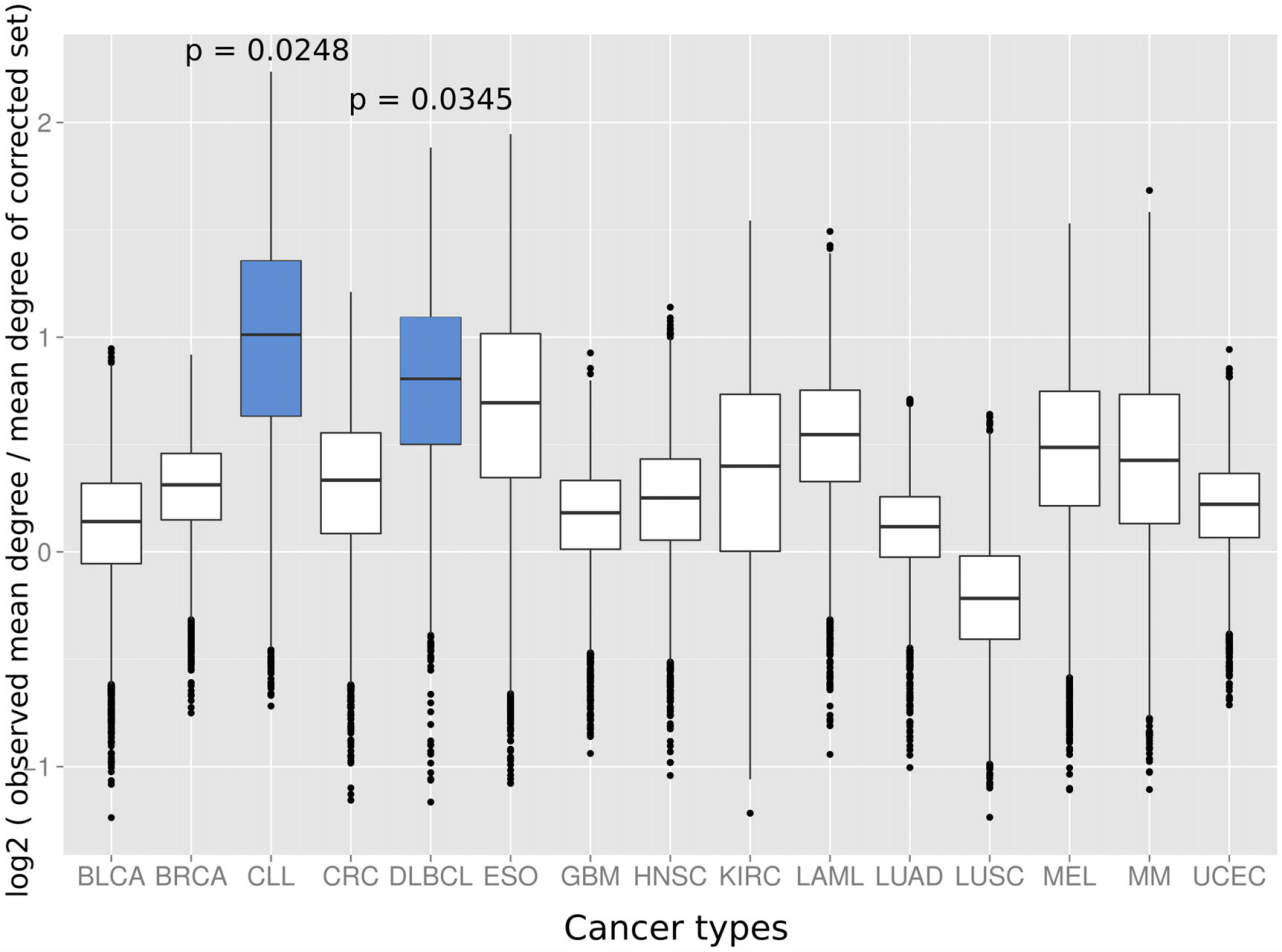
**The distributions show the log2 of the mean degree of proteins of the specific cancer type divided by the mean degree of 10,000 randomized protein sets with the same bait usage distribution (a value of 0 would therefore signify that the mean degree of the cancer protein set equals the observed mean degree of the random protein set, positive values that the mean degree of the proteins of the respective cancer type is higher than for the random set and, vice versa, negative values that the mean degree of the random set proteins is higher as for the proteins of the cancer type).** Blue boxes indicate that the mean of the original degree distribution of the respective cancer type is significantly higher (*p* < 0.05; randomization test) as those of randomized protein sets with the same bait usage distribution. The cancer types on the *x*-axis are: BLCA, bladder cancer; BRCA, breast cancer; CLL, chronic lymphocytic leukemia; CRC, colorectal cancer; DLBCL, diffuse large B-cell lymphoma; ESO, esophageal adenocarcinoma; GBM, glioblastoma multiforme; HNSC, head and neck cancer; KIRC, kidney clear cell carcinoma; LAML, acute myeloid leukemia; LUAD, lung adenocarcinoma; LUSC, lung squamous cell carcinoma; MEL, melanoma; MM, multiple myeloma, and UCEC, endometrial cancer.

Next we investigated if the deviation between observed and expected degree distributions differs across cancer types. Therefore, we applied the same randomization strategy as before to correct the study bias in the degree distributions of cancer proteins from 15 different tumor types (Lawrence et al., 2014). An interesting picture emerged: while proteins from several cancer types had close to random expectation degree distributions, most cancers of the hematological system had the highest deviation between mean of the observed degree distribution and the mean of the degree distribution of randomly sampled protein sets studied similarly often for interaction partners (**Figure 3**). The highest deviations between observed and expected degree distribution were for chronic lymphocytic leukemia (CLL; *p* = 0.0248; randomization test), diffuse large B-cell lymphoma (DLBCL; *p* = 0.0354; randomization test) and acute myeloid leukemia (LAML; *p* = 0.0525; randomization test). Interestingly, the higher degree distribution of hematological cancer proteins is achieved by distinct protein sets and not an artifact of overlapping cancer protein sets: no protein was associated to these three cancers and just three proteins appeared in association with two (see **Supplementary Table S1**).

To investigate possible functional reasons for the higher than expected by chance degree distribution of hematological cancer proteins, we computed for the proteins from those cancer classes the ratio between the degree and the number of times a protein had been tested as a bait protein (as a proxy for a bias normalized degree estimate; **Supplementary Table S1**). For each of the three cancer types, we focused on the 50% of the proteins with the highest ratio. Interestingly, in two of the cases the most highly connected proteins were indicative of cancerogenesis processes specific to the respective hematological tumor.

Two (RPS15 and XPO1) of the three CLL proteins with the highest ratio of degree to experiments (out of six CLL proteins for which we have PPI experimental data) are involved in the establishment of ribosome localization (while none are from the proteins with a lower ratio). The third of the highest ratio proteins (SF3B1) is also a ribonucleoprotein. The higher degree of the ribosome-related proteins (*p* = 0.05; Fisher test) is not surprising as 100s of closely interacting proteins are involved in the biogenesis and transport of the ribosomal subunits (Fromont-Racine et al., 2003; Altvater et al., 2012). Interestingly, CLL cells show impaired assembly of ribosomes (Rubin, 1971), which likely reduces their metabolic activity and helps them to avoid cell death (Defoiche et al., 2010).

Of the seven DLBCL proteins with the highest ratio, five were involved in the activation of leukocytes (of a total of 13 DLBCL proteins with bait usage information). From the six proteins with lower ratio none was associated with this function (*p* < 0.05; Fisher test). Interestingly, many lymphomas resemble gene expression patterns of activated B cells (Alizadeh et al., 2000). Leukocyte activation has been shown to be driven by a large and highly interconnected protein network (Calvano et al., 2005).

The examples of ribonucleoproteins in CLL and leukocyte activators in DLBCL illustrate how selection for tumor-specific functions modify the observed degree distribution of each tumor. In conclusion, there is no generally elevated connectivity of cancer proteins. Only in some cancer types groups of proteins tend to be mutated that belong to highly interconnected cellular networks.

To estimate how robust our observations are with respect to variations in the computation of the test statistic, we repeated the randomization procedure computing the median degree of the original and randomized protein sets instead of the mean. The overall observation remained unchanged: random proteins with bait usage similar to that of cancer proteins have higher degree than random proteins without any constraints on the bait usage (both for mean and median; **Supplementary Figure S2**). However, using the median we observed a significant degree enrichment for cancer proteins (*p* < 0.01; randomization test) and this time CLL, LAML, and BRCA had significantly higher number of PPIs as compared to random sets (all *p* < 0.05; randomization test).

## Discussion

Here, we quantify how the frequency with which a protein has been studied for interaction partners affects its reported degree distribution. We estimate that the resulting bias is higher than previously quantified biases resulting from technical limitations. For example, the correlation between protein abundance and degree ranges for different TAP/MS networks from 0.21 to 0.46 (Ivanic et al., 2009) while we observe a correlation >0.5 between the number of times a protein has been tested as a bait and its degree.

Our findings have a dramatic impact on the common understanding of the relation between protein function and degree. Specifically, we challenge the previous finding that cancer proteins tend to have more interaction partners than non-cancer proteins (Wachi et al., 2005; Jonsson and Bates, 2006; Rambaldi et al., 2008). In fact a more complex picture emerges: while some cancer types are associated with proteins of lower or average connectivity, others are associated with promiscuous proteins. The different degree distributions correlate with functional specificities of the tumor types. Interestingly, the higher degree distribution of hematological cancer genes is driven by largely different protein sets with distinct functions (the proteins with the highest ratio between degree and bait usage are ribonucleoproteins for CLL and proteins involved in leukocyte activation for DLBCL).

From our analysis it follows that many cancer gene prediction approaches might have overestimated their performance as they directly or indirectly use the PPI degree as a feature for classification. A classifier that preferentially selects proteins with high degree will therefore favor highly studied proteins, which in turn are more likely to be cancer proteins. This should be taken into consideration by either using less biased networks from proteome-scale screens or by omitting degree-related features for classification.

One limitation of the presented method is that the reported number of times a protein has been tested as a bait gives only a rough and a lower bound estimate as for many experiments this information is not available in the public databases. Also, the distinction between bait and prey protein might not apply to all types of experimental methods (as for example for crystallization of complexes). As described in the Results section, our method shows a certain sensitivity with respect to the chosen statistics. However, the overall tendency in the results stayed the same when the median instead of the mean was computed: randomly sampled proteins that have been studied as often as cancer proteins are more similar in their degree distribution to cancer proteins as to arbitrarily often studied proteins.

In summary, we argue for the crucial importance of taking into account the number of times a protein has been studied when analyzing PPI networks. Ignoring the resulting degree distribution bias is not just leading to wrong biological assumptions on the relation between network topology and protein function but also introduces circularity into network-based disease gene prediction.

To come to reliable conclusions regarding degree differences between protein classes, it would be generally favorable if rarely studied proteins would be increasingly often tested for PPI partners to eliminate the differences in the very uneven bait usage distribution. While these sharp differences persist, the here presented methods can help to reduce the impact of the study bias when comparing degree distributions and could be applied to other disease protein classes.

## Author Contributions

MS and MA conceived and designed the analyses. MS analyzed the data. MS, LS, and MA wrote the paper.

## Conflict of Interest Statement

The authors declare that the research was conducted in the absence of any commercial or financial relationships that could be construed as a potential conflict of interest.

## Supplementary Material

FIGURE S1 **The number of studies in which a protein has been tested as a bait for interaction partners is plotted against the number of reported interactions in linear scale**.

FIGURE S2 **We randomly sampled 100 protein sets of size 10 (a) from the cancer proteins, (b) equally often studied (as bait) non-cancer proteins, and (c) non-cancer proteins without any constraints on the bait usage**. We computed both the mean and the median for each of the resulting 300 protein sets. The resulting mean/median degree distributions are shown. Although with this sampling strategy all distributions are pairwise dissimilar (^∗^*p* < 0.05; ^∗∗^*p* < 0.01; ^∗∗∗^*p* < 0.001), the random proteins that have been studied as often as the cancer proteins have a much more similar degree distribution to the cancer proteins as compared to randomly sampled background proteins (even though the similarity is higher when the mean is computed than when the median is computed).

TABLE S1 **S1 The table shows hematological cancer proteins for which PPI and bait usage information was available**. Gene name and entrez gene id, tumor type and the ratio between the degree and the number of times the protein has been tested as a bait are indicated.
