# Supplementary figures and images for: Correcting for the study bias associated with protein–protein interaction measurements reveals differences between protein degree distributions from different cancer types
Source: Front Genet. 2015 Aug 4;6:260. doi: 10.3389/fgene.2015.00260 (PMC4523822; doi:10.3389/fgene.2015.00260)

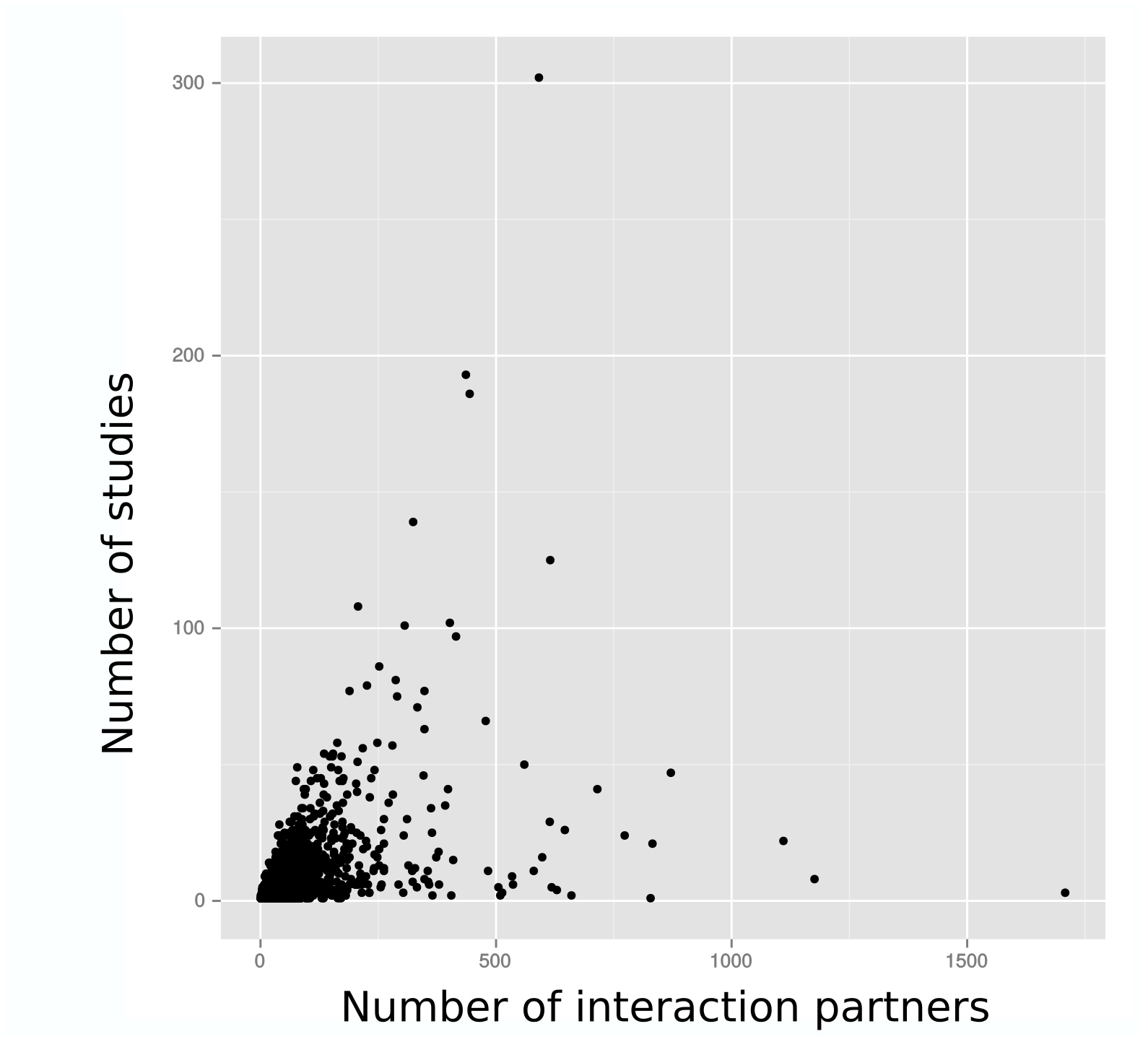

Supplement: FIGURE S1 — The number of studies in which a protein has been tested as a bait for interaction partners is plotted against the number of reported interactions in linear scale. [file Image_1.JPEG]

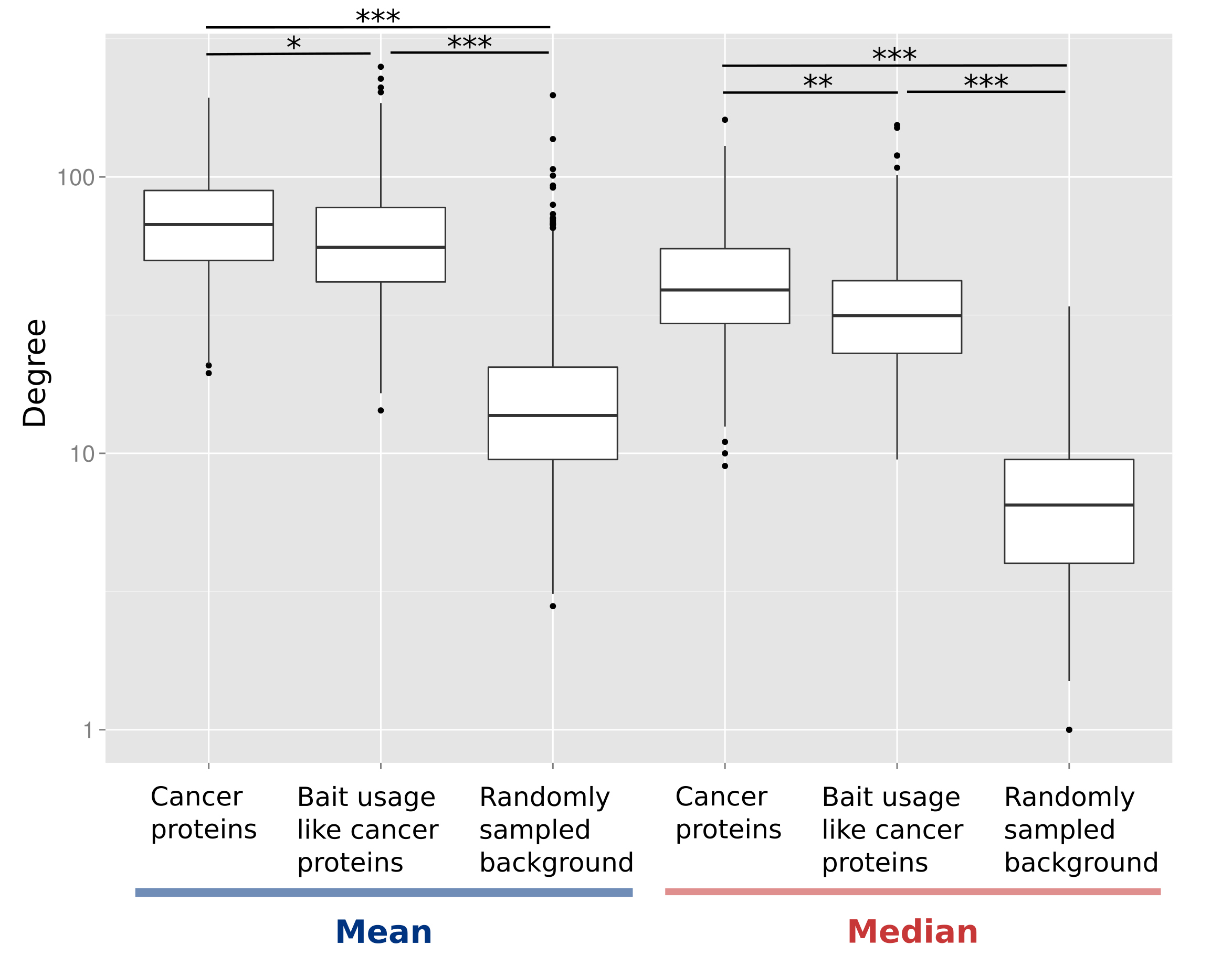

Supplement: FIGURE S2 — We randomly sampled 100 protein sets of size 10 (a) from the cancer proteins, (b) equally often studied (as bait) non-cancer proteins, and (c) non-cancer proteins without any constraints on the bait usage. We computed both the mean and the median for each of the resulting 300 protein sets. The resulting mean/median degree distributions are shown. Although with this sampling strategy all distributions are pairwise dissimilar (∗p < 0.05; ∗∗p < 0.01; ∗∗∗p < 0.001), the random proteins that have been studied as often as the cancer proteins have a much more similar degree distribution to the cancer proteins as compared to randomly sampled background proteins (even though the similarity is higher when the mean is computed than when the median is computed). [file Image_2.JPEG]
